# Clinical efficacy and complications of transurethral resection of the prostate versus plasmakinetic enucleation of the prostate

**DOI:** 10.1186/s40001-023-00989-9

**Published:** 2023-02-18

**Authors:** Chong-Yi Yang, Ge-Ming Chen, Yue-Xiang Wu, Wei-Jie Zhang, Jie Wang, Peng-Peng Chen, Zhen-Yuan Lou

**Affiliations:** 1 Department of Urology, Ninghai First Hospital, Ningbo, 315600 Zhejiang China; 2 Department of Urology, The First Affiliated Hospital of Zhejiang University, Hangzhou, 310000 Zhejiang China; 3 Community Health Service Center of Yuehu, Ningbo, 315000 Zhejiang China

**Keywords:** Transurethral resection of the prostate, Plasmakinetic enucleation of the prostate, Benign prostatic hyperplasia, Complications

## Abstract

**Background:**

Benign prostatic hyperplasia (BPH) is a common disease in elderly males, and many kinds of minimally invasive procedures can be used for the treatment of BPH. However, various procedures have caused some controversies regarding clinical outcomes, so more studies are needed to validate these controversial topics.

**Aims:**

This study aimed to explore differences of clinical efficacy, surgical features, and complications between transurethral resection of the prostate (TURP) and plasmakinetic enucleation of the prostate (PKEP) for BPH.

**Methods:**

A total of eligible 850 cases of BPH underwent TURP (the TURP group, 320 cases) or PKEP (the PKEP group, 530 cases) in the urology department of our hospital from March 2015 to 2018 were involved in this study. Then, the baseline data, surgical characteristics, IPSS, QoL, PVR, Q_max,_ IIEF-5, and documented complications were compared between the two groups.

**Results:**

The operative time, intraoperative irrigation volume, postoperative hemoglobin, decrease in hemoglobin, postoperative irrigation time and volume, catheterization time, and hospital stay of the PKEP group were significantly less than those of the TURP group (all *P* < 0.05). At 3 months, 1, 2, and 3 years after operation, no significant differences were observed in IPSS, QoL, PVR, but the results of Q_max_ and IIEF-5 in the PKEP group were significantly higher than those parameters in the TURP group (all *P* < 0.05). The incidences of massive blood loss, postoperative secondary bleeding, blood transfusion, capsular perforation, urinary tract irritation, bladder spasm, clot retention, urinary tract infection, transient incontinence, erectile dysfunction, and the incidences of II, III grade of Clavien–Dindo classification in the PKEP group were significantly lower than those of the TURP group (all *P* < 0.05).

**Conclusion:**

The clinical efficacy of PKEP is compared favorably with TURP during midterm follow-up. Given the merits such as less blood loss and hospital stay, lower complications, PKEP should be given a priority for BPH.

## Introduction

Benign prostatic hyperplasia (BPH) is a histologic diagnosis describing epithelial cells and smooth muscle proliferation within the prostatic transition area. It is a common disease with lower urinary tract symptoms in middle-aged and elderly males, which is becoming more prevalent in parallel with the aging of demographic structures in China [1]. In recent years, the overall morbidity of elderly males was approximately 50%, and the prevalence of BPH is about 70% in men over 70 years old [2–4]. There are many medical and surgical treatment choices for BPH, such as medical drugs, traditional Chinese medicine, physiotherapeutic techniques, surgical approaches, and combination therapy [5, 6]. When these conservative therapies were invalid for BPH, surgical approaches were considered necessarily. Transurethral resection of the prostate (TURP) had always been considered the “gold standard” because of its well-documented efficacy and low-cost burden in the past decades [7]. Nowadays, improvements in medical equipments and techniques have made surgical choices become to more diverse for BPH, such as TURP, plasmakinetic enucleation of the prostate (PKEP), laser enucleation (holmium laser, thulium laser, green laser), photoselective vaporization of the prostate and other approaches. However, so many surgical treatments have made some discrepancies and controversies regarding their complications, safety profile, and sexual function among these surgical approaches [8, 9].

In the past twenty years, TURP and PKEP were the two main types of surgical approaches in our hospital, many previous studies had been reported about their comparisons on clinical efficacy, and most of researchers considered that PKEP and TURP had a similar clinical efficacy. Nevertheless, controversies regarding surgical features, complications, and sexual function have never stopped, so more studies should be performed to update and validate these debatable topics [10]. For these reasons, we collected 850 eligible cases of BPH who underwent PKEP or TURP in our hospital from March 2015 to March 2018. Then, an observation study was performed to assess the differences of complications, safety profile, sexual function, and other concerns between TURP and PKEP.

## Patients and methods

### Patients

**Fig. 1:**
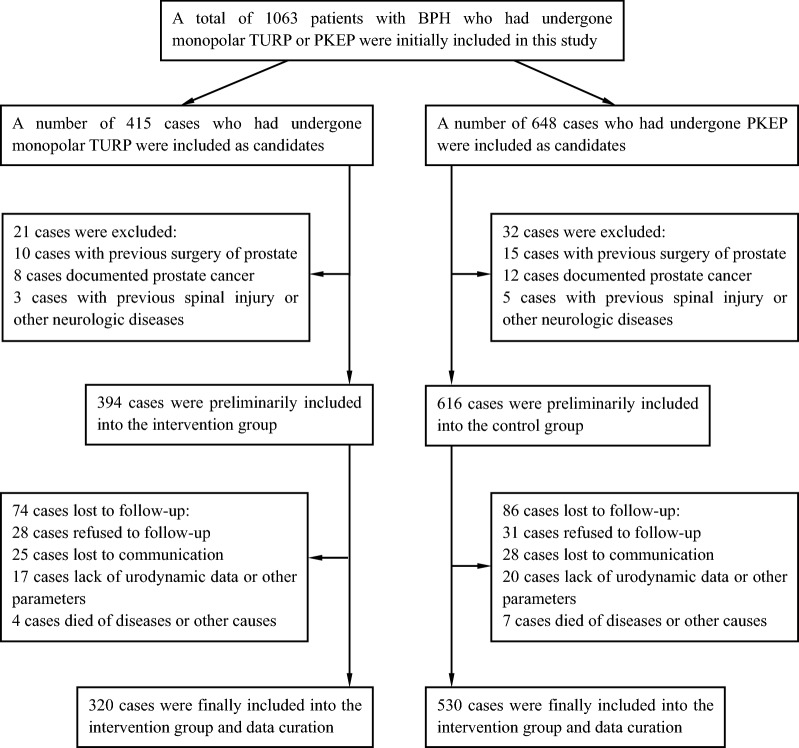
Flowchart of patients in this study

A total of 1063 patients with BPH who had undergone monopolar TURP or PKEP from March 2015 to March 2018 were initially included in this study. According to surgical approaches, 415 cases underwent monopolar TURP were included as candidates in the TURP group, and 648 cases underwent PKEP were included as candidates in the PKEP group. Inclusion criteria included patients who were diagnosed with BPH in clinic and pathological findings, and invalid for rigorously conservative treatments, as well as the age of these patients were more than 50 years. Exclusion criteria included patients with previous surgery of prostate, documented prostate cancer, and previous spinal injury or other neurologic diseases. According to the criteria mentioned above, 21 cases were excluded in the TURP group, and 32 cases were excluded in the PKEP group. During three years of follow-up, 14, 21, and 39 cases in the TURP group and 17, 24, and 45 cases in the PKEP group at 1, 2, and 3 years were, respectively, excluded due to loss to follow-up or other causes. Finally, 320 cases in the TURP group and 530 cases in the PKEP group were retained in this study. The flowchart of patients in this study is presented in Fig. 1.

### Surgical approaches

Before operation, patients with comorbidities (including hypertension, Diabetes, coronary heart disease) included in this study should be treated until patients are allowed to receive operation. All patients in the two groups received spinal anesthesia and were placed in a lithotomy position, and both procedures were performed by one of two experienced surgeons (CY or GC). The monopolar TURP was performed using the Wolf F_27_ resectoscope (Wolf, Germany) with power output set at 100 ~ 140 W for resection and 60 ~ 80 W for coagulation. The PKEP was performed using the F_27_ resectoscope of Gyrus plasmakinetic system (Gyrus Medical, UK) with a cutting power of 140 ~ 160 W and a coagulating power of 80 ~ 100 W. The 5% glucose solution was used for irrigation in monopolar TURP, and 0.9% saline solution was used for irrigation in PKEP. The methods of the two procedures were similar as in the previous study, which was described by Cai F et al. [11]. The operation was carried out from apical tissue towards the neck, followed by resection of the anterior lobe and lateral lobes, deep into the prostate capsule. Any hemorrhagic sources should be careful to coagulate, and the excised tissues should be washed out. A 22 F Foley catheter was inserted into the bladder, and followed by irrigation. The Foley catheters were removed when hematuria ceased and the effluent fluid was completely clear.

### Study variables

Preoperative data (including age, duration of BPH, volume of prostate, comorbidities, serum PSA, etc.) were obtained from our medical records, and perioperative data (including operating time, irrigation volume and time, decrease in hemoglobin, catheterization time, hospital stay, etc.) were also recorded. Complications or adverse events (including massive blood loss, postoperative secondary bleeding, blood transfusion, capsular perforation, bladder injury, etc.) in the two groups were recorded during perioperative or follow-up, and each complication was classified by the I~ Vgrade with referring to the Clavien–Dindo classification [12]. The resected tissue weight was evaluated according to the actual weight of tissue retrieved, and postoperative hemoglobin level was detected within 24 h after the operation. Urinary infection were diagnosed by urine culture, and retrograde ejaculations were defined as urine sample with a large number of sperms after ejaculation [13]. Each enrolled patient received an assessment in the International Prostate Symptom Score (IPSS), Quality of life score (QoL), Maximum flow rate (Q_max_), Postvoid residual urine (PVR) at the preoperation and 3 months, 1, 2, 3 years of follow-up. The PVR and prostatic volume were measured by abdominal ultrasonography or transrectal ultrasound, respectively. Sexual function was evaluated by the International Index of Erectile Function (IIEF-5), and the score of IIEF-5 with less than 12 points was considered as typical erectile dysfunction [14]. During the course of follow-up, if a patient with one time of the absence in IPSS, QoL, Qmax, PVR, and IIEF-5, the absent value of a parameter can be filled by imputation or mean completer at the observation time of 1, 2, and 3 years. If a patient with more than two times of the absence in these parameters, the patient was also considered as the loss to follow-up.

### Statistical analysis

Statistical analysis of data was performed using SPSS 22.0. The numerical data were given as mean $\pm$ standard deviation (${\overline{\text{x}}}$ ± SD), and analyzed with *t* test. The categorical data were given as percentage (%), and analyzed with *x*^*2*^ test or fisher’s exact test. *P* < 0.05 was considered as significance in statistics.

## Results

### Comparisons of baseline data

For comparisons of baseline data before operation, no significant differences were observed between the TURP group and the PKEP group (all *P* > 0.05) (Table 1).

**Table 1:** Comparisons of baseline data in the two groups [(${\overline{\text{x}}}$_±_SD), n(%)]

Parameters	TURP group (320)	PKEP group (530)	*t or x*^*2*^	*P*
Age (year)	67.94 ± 10.16	68.75 ± 8.97	1.213	0.226
Duration of BPH (year)	9.89 ± 3.24	_10.11±4.06_	_0.882_	_0.411_
Volume of prostate (mL)	62.23 ± 19.25	60.44 ± 17.79	1.362	0.173
Catheterization (*n*)	51(15.94)	69(13.02)	1.049	0.306
Bladder stone (*n*)	45(16.06)	52(9.81)	2.811	0.094
Hypertension (*n*)	86(26.88)	163(30.75)	1.450	0.229
Diabetes (*n*)	75(23.44)	102(19.25)	1.382	0.240
Coronary heart disease (*n*)	63(19.69)	115(21.70)	0.487	0.485
Serum tPSA (ng/L)	_2.07±0.82_	_1.98±0.75_	1.636	0.102
VAS score (points)	_2.63±0.61_	_2.56±0.41_	1.300	0.235
Erectile dysfunction (*n*)	89(27.81)	163(30.75)	0.828	0.363
Retrograde ejaculation (*n*)	25(7.81)	39(7.36)	0.051	0.822
ASA classification (I ~ II)	231(72.19)	408(76.98)	0.351	0.554
ASA classification (III ~ IV)	89(27.81)	128(24.15)	0.829	0.363

*TURP* transurethral resection of prostate; *PKEP* plasmakinetic enucleation of the prostate; *tPSA* total prostate specific antigen; *ASA* the American Society of Anesthesiologists

### Comparisons of perioperative data

The operative time, intraoperative irrigation volume, postoperative hemoglobin, decrease in hemoglobin, postoperative irrigation time and volume, catheterization time, and hospital stay of the PKEP group were significantly less than those in the TURP group (all *P* < 0.05) (Table 2).

**Table 2:** Comparisons of perioperative data in the two groups (${\overline{\text{x}}}$_±_SD)

Parameters	TURP group (320)	PKEP group (530)	*t*	*P*
Operative time (min)	69.95 ± 16.93	56.29 ± 15.77	10.157	< 0.001
Intraoperative irrigation volume (L)	15.26 ± 3.52	12.38 ± 3.24	12.151	< 0.001
Weight of resected prostate (g)	48.65 ± 15.21	46.89 ± 14.57	1.678	0.094
Preoperative hemoglobin (g/L)	133.67 ± 13.64	134.25 ± 14.91	1.545	0.123
Postoperative hemoglobin (g/L)	124.01 ± 12.75	127.13 ± 13.52	3.330	0.001
Decrease in hemoglobin (g/L)	9.66 ± 3.05	7.13 ± 2.46	13.250	< 0.001
Postoperative irrigation time (d)	2.03 ± 0.51	1.79 ± 0.46	6.894	< 0.001
Postoperative irrigation volume (L)	24.45 ± 6.64	19.62 ± 4.78	11.356	< 0.001
Catheterization time (d)	2.46 ± 0.78	2.28 ± 0.58	3.574	0.009
VAS score (3 days after operation)	_3.21±1.05_	_3.27±0.97_	1.694	0.091
Hospital stay (d)	5.19 ± 1.50	4.33 ± 1.34	8.663	< 0.001

*TURP* transurethral resection of prostate; *PKEP* plasmakinetic enucleation of the prostate; *VAS* Visual analogue scale

### Comparisons of IPSS, QoL, Qmax, PVR, and IIEF-5

There were no significant differences between the two groups in preoperative IPSS, QoL, Q_max_, and PVR. At the 3 months, 1, 2, and 3 years of follow-up, the IPSS, QoL, Q_max_, PVR were both obviously improved in the two groups, and no significant differences were observed in IPSS, QoL, PVR between the two groups (all *P* > 0.05), but the results of Q_max_ and IIEF-5 in the PKEP group were significantly higher than those parameters in the TURP group (all *P* > 0.05) (Table 3).

**Table 3:** Comparisons of IPSS, QoL, Q_max_, PVR, and IIEF-5 in the two groups (${\overline{\text{x}}}$_±SD_)

Group	*n*	pre-operation	3 months	1 year	2 years	3 years
IPSS						
TURP group	320	24.03 ± 4.66	7.20 ± 2.48	6.22 ± 2.31	6.65 ± 2.02	7.20 ± 2.53
PKEP group	530	24.34 ± 5.11	6.95 ± 2.26	5.94 ± 2.18	6.46 ± 1.98	6.99 ± 2.31
* t*		0.885	1.506	1.774	1.345	1.238
* P*		0.386	0.132	0.076	0.179	0.216
QoL						
TURP group	320	4.60 ± 0.73	1.41 ± 0.43	1.05 ± 0.33	1.33 ± 0.72	1.60 ± 0.62
PKEP group	530	4.69 ± 0.82	1.37 ± 0.39	1.10 ± 0.42	1.27 ± 0.48	1.54 ± 0.57
*t*		1.662	1.393	1.927	1.324	1.438
*P*		0.141	0.164	0.095	0.227	0.151
Q_max_ (mL/s)						
TURP group	320	7.50 ± 1.94	25.36 ± 7.24	23.75 ± 5.40	24.12 ± 6.28	22.93 ± 4.48
PKEP group	530	7.66 ± 2.03	26.48 ± 6.93	25.64 ± 4.58	25.33 ± 5.54	24.70 ± 4.85
* t*		1.132	2.245	5.443	2.932	5.304
* P*		0.258	0.025	< 0.001	0.003	< 0.001
PVR (mL)						
TURP group	320	98.85 ± 20.47	19.66 ± 5.05	20.45 ± 6.68	22.27 ± 7.45	24.32 ± 8.09
PKEP group	530	101.37 ± 25.18	19.00 ± 4.89	19.70 ± 7.04	21.68 ± 6.84	23.78 ± 7.55
* t*		1.718	1.883	1.534	1.178	0.983
* P*		0.103	0.060	0.125	0.239	0.326
IIEF-5						
TURP group	320	17.26 ± 4.28	16.50 ± 3.94	17.26 ± 3.25	16.78 ± 4.16	15.77 ± 4.75
PKEP group	530	16.81 ± 3.86	17.20 ± 4.13	_18.03±3.47_	_17.61±3.83_	16.89 ± 4.28
* T*		1.540	2.436	3.209	2.963	3.545
* P*		0.167	0.015	0.001	0.003	< 0.001

*TURP* transurethral resection of prostate; *PKEP* plasmakinetic enucleation of the prostate; *IPSS* international prostate symptom score; *QoL* quality of life; *Q*_*max*_ maximum flow rate; *PVR* postvoid residual urine; *IIEF-5* International Index of Erectile Function 5

### Comparisons of complications or adverse events

The incidences of massive blood loss, postoperative secondary bleeding, blood transfusion, capsular perforation, urinary tract irritation, bladder spasm, clot retention, urinary tract infection, transient incontinence, and erectile dysfunction (increased cases after operation) of the PKEP group were significantly lower than those of the TURP group (all *P* < 0.05), and the incidences of II, III grade in the PKEP group were significantly lower than those of the TURP group (all *P* < 0.05) (Table 4).

**Table 4:** Comparisons of complications or adverse events in the two groups [n(%)]

Parameters	TURP group (320)	PKEP group (530)	*x*^*2*^	*P*
Massive blood loss (> 600 mL)	15(4.69)	9(1.70)	6.499	0.011
Postoperative secondary bleeding	21(6.56)	7(1.32)	17.209	< 0.001
Blood transfusion	14(4.38)	6(1.13)	9.133	0.003
Capsular perforation	6(1.88)	2(0.75)	4.800	0.028
Bladder injury	1(0.43)	0(0.00)	–	0.798
Urinary tract irritation	109(34.06)	117(22.08)	14.688	< 0.001
Bladder spasm	32(10.00)	29(5.47)	6.142	0.013
Clot retention	13(4.06)	4(0.75)	11.138	0.001
Acute epididymitis	3(0.94)	4(0.75)	0.011	0.916
Urinary tract infection	10(3.13)	6(1.13)	4.291	0.038
Shock	4(1.25)	2(0.38)	1.102	0.294
Transurethral resection syndrome	1(0.43)	0(0.00)	–	0.129
Transient incontinence	17(5.31)	12(2.26)	5.626	0.018
Permanent incontinence	0(0.00)	2(0.38)	–	0.351
Urethral stricture	8(2.50)	6(1.13)	2.305	0.129
Erectile dysfunction ^#^	84(26.25)	96(18.11)	7.914	0.005
Retrograde ejaculation ^#^	119(37.19)	167(31.51)	2.881	0.090
Recurrence	3(0.94)	2(0.38)	0.327	0.567
Clavien–Dindo Classification ^△^				
I	164(51.25%)	243(45.85%)	2.332	0.127
II	83(25.94%)	87(16.42%)	11.308	0.001
III	19(5.94%)	13(2.45%)	6.688	0.010
IV	5(1.56%)	3(0.57%)	2.125	0.145
V	1(0.31%)	0(0.00%)	–	0.798

*TURP* transurethral resection of prostate; *PKEP* plasmakinetic enucleation of the prostate

^a^indicated that increased cases after operation

^b^If a patient have two or more kinds of complications, the most severe complication was defined as the grade of Clavien–Dindo classification

## Discussion

For many years ago, open prostatectomy had been the primary option in BPH patients until it was gradually replaced by TURP. Since then, TURP has been applied in the treatment of BPH for decades, which was recognized as the “golden standard” owing to its clinical efficacy [15]. In previous studies, many researchers have discussed the differences of TURP compared with other surgical procedures, including clinical efficacy, safety profile, sexual function, and so on, but there was no consensus about these topics [16, 17]. In this study, only Q_max_ appeared to have a significant difference with the minor gap, and no significant differences were observed in IPSS, QoL, and PVR between the two groups during the follow-up. The results of our study demonstrated that TURP has comparable clinical efficacy in contrast to PKEP, and these findings in IPSS, QoL and PVR were similar to previous studies performed by others [18–20].

In this study, the operative time, intraoperative irrigation volume, postoperative hemoglobin, decrease in hemoglobin, postoperative irrigation time and volume, catheterization time, and hospital stay of PKEP were significantly less than those of TURP (*P* < 0.05), which indicated that PKEP has some merits in operation time, irrigation time and volume, catheterization time, blood loss, and hospital stay [21]. To compare the differences of complications or adverse events in the two groups, about 20 parameters were enrolled in this study, and our results showed that the incidences of massive blood loss, postoperative secondary bleeding, blood transfusion, capsular perforation, urinary tract irritation, bladder spasm, clot retention, urinary tract infection, transient incontinence, and erectile dysfunction of the PKEP group were significantly lower than those of the TURP group (*P* < 0.05). These findings suggested that PKEP has less complication and better safety profile, and this viewpoint was also supported by a study reported by Li S et al. [22]. The Clavien–Dindo classification was widely used in many surgical reports, which was a 5-scale classification of surgical complications based on the type of treatment for complications [23]. We used it for grade assessment of complications, and our results showed that the incidences of II, III grade in the Clavien–Dindo classification of PKEP were significantly lower than those of TURP (*P* < 0.05), which also suggested that PKEP have better safety profile compared with TURP.

At the early stage of development in TURP technique, transurethral resection syndrome was one of the most serious complication of TURP. In recent years, improvements in surgical skills and medical technology made transurethral resection syndrome very rare. In this study, the incidence of transurethral resection syndrome was 0.43% in the TURP group. One patient suffered from transurethral resection syndrome in the TURP group in contrast to none in the PKEP group. In PKEP, 0.9% saline solution was used for irrigation, and it can significantly reduce the risk of dilutional hyponatremia and transurethral resection syndrome. In addition, vaporized resection of PKEP can generate coagulation at the tissue interface, which will make a quick close for small blood vessels and capillaries, consequently preventing reabsorption of the irrigation solution [24]. Moreover, PKEP using a bipolar plasmakinetic system can achieve cutting and coagulation simultaneously with a plasma-loop electrode. Compared with satisfactory hemostasis of PKEP, some conditions caused by more blood loss in TURP will increase the incidence of hemorrhage-related complications. In this study, we also considered that the differences in blood loss and blood transfusion between the two groups were related to this coagulation and hemostasis.

A previous study considered that the good hemostasis of PKEP can shorten the catheterization and hospitalization time [25]. In the view of results in our study, the endoscopic vision and lower cutting temperature in PKEP technique should be taken into account. Firstly, a clear view offered by better hemostasis not only can shorten operation time and irrigation volume but also reduce complications and iatrogenic injury such as capsular perforation and bladder injury. Secondly, the high loop-cutting temperature of TURP will result in thermal injury and wound scarring. When the scar was dropped out during wound self-healing, subsequently leads to secondary hemorrhage after operation, which also increases the catheterization time and hospital stay. Moreover, the temporary urinary tract irritation after operation in TURP may be caused by thermal injury, thermal-related inflammation, and tissue edema [26, 27]. Hence, a higher incidence of urinary tract irritation in TURP was observed compared with PKEP. There were two patients who suffered from permanent incontinence in PKEP, and the specific cause was not very clear, which may be related to injury of urethral sphincter or diabetic neurogenic bladder dysfunction [28].

In this study, the postoperative IIEF-5 score of the PKEP group was significantly higher than that of the TURP group, and the incidence of erectile dysfunction (increased cases after operation) in the PKEP group was significantly lower than that of the TURP group. During the operation, PKEP can offer a clear visual field and reduce probability of nerve injury, and it will alleviate the negative impact on sexual function, which may be responsible for better sexual function in PKEP. On the other hand, some dissimilarities among psychological aspects, sexual culture and concept from different areas should be taken into account, most Chinese elder people were ashamed of facing sex and related topics, and some patients’ obscure responses toward the survey may become a confounding factor to interpret these results objectively. Presently, the change in sexual function after operation is a debatable topic among studies from different areas and countries [17, 29]. Therefore, more studies regarding this discrepancy are required for a definite conclusion.

In our study, some limitations should be presented here. Firstly, at the beginning of this study, the randomized controlled design was planned to apply, but too many patients after allocation had made an unexpected alterations such as canceling in operation, asking for another procedure, or retreating in this study, and it's challenging for us to perform a randomized controlled trial of surgical procedures in this sample size. Ultimately, we only grouped by patients’ final procedures and made a comparative analysis by means of obtained patients’ data, which will dilute its referential merits in some way. Secondly, in the later stage of this study, patients always had a tendency to choose PKEP due to its merits in terms of less hospital stay, lower complications, and more comfort. Then, it resulted in a not even distribution of cases in each group (320 cases in TURP versus 530 cases in PKEP). Thirdly, some patients during three years of follow-up had once absence of IPSS, QoL, Qmax, PVR, or IIEF, and the absent value of one parameter was allowed to be filled by imputation except for the preoperative evaluation and the first postoperative evaluation. After that, a good retention of cases was presented in this study during follow-up. Nevertheless, it can generate a mild bias in the results even though the total of cases was no more than 30% in whole samples. In view of limitations mentioned above, more high-quality study with evidence-based medicine needs to be performed in the future.

To summarize, the clinical efficacy of PKEP is compared favorably with TURP during three years of follow-up, but PKEP has advantages in less thermal injury and blood loss, lower complications, and better safety profile, which may be more suited for high-risk patients such as cardiac pacemaker implantation or people taking anticoagulant / antithrombotic drugs [30–32]. In conclusion, PKEP should be given a priority for BPH surgical treatment. However, in consideration of limitations in this study, our results need to be confirmed in well-designed and multicentre randomized controlled trials.

## Data Availability

The datasets used and/or analyzed can be accessed from the corresponding author on reasonable request.
